# Inhibitory Effect of Plant *Manilkara subsericea* against Biological Activities of *Lachesis muta* Snake Venom

**DOI:** 10.1155/2014/408068

**Published:** 2014-01-08

**Authors:** Eduardo Coriolano De Oliveira, Caio Pinho Fernandes, Eladio Flores Sanchez, Leandro Rocha, André Lopes Fuly

**Affiliations:** ^1^Laboratório de Venenos e Toxinas de Animais e Avaliação de Inibidores, Departamento de Biologia Celular e Molecular, Instituto de Biologia, Universidade Federal Fluminense, Outeiro de São João Batista, 3**º** andar, Sala 310, 24020-141 Niterói, RJ, Brazil; ^2^Programa de Pós-Graduação em Ciências Aplicadas a Produtos para a Saúde, Faculdade de Farmácia, Universidade Federal Fluminense, 24220-900 Niterói, RJ, Brazil; ^3^Programa de Pós-Graduação em Biotecnologia Vegetal, Centro de Ciências da Saúde, Universidade Federal do Rio de Janeiro, 21941-599 Rio de Janeiro, RJ, Brazil; ^4^Centro de Pesquisa e Desenvolvimento, Fundação Ezequiel Dias, 30510-010 Belo Horizonte, MG, Brazil

## Abstract

Snake venom is composed of a mixture of substances that caused in victims a variety of pathophysiological effects. Besides antivenom, literature has described plants able to inhibit injuries and lethal activities induced by snake venoms. This work describes the inhibitory potential of ethanol, hexane, ethyl acetate, or dichloromethane extracts and fractions from stem and leaves of *Manilkara subsericea* against *in vivo* (hemorrhagic and edema) and *in vitro* (clotting, hemolysis, and proteolysis) activities caused by *Lachesis muta* venom. All the tested activities were totally or at least partially reduced by *M. subsericea*. However, when *L. muta* venom was injected into mice 15 min first or after the materials, hemorrhage and edema were not inhibited. Thus, *M. subsericea* could be used as antivenom in snakebites of *L. muta*. And, this work also highlights Brazilian flora as a rich source of molecules with antivenom properties.

## 1. Introduction

Snakebites are a public health problem that affects mainly poor people who live in rural areas of Africa, Asia, Latin America, and Oceania [1, 2]. According to World Health Organization, it is estimated that up to five million people are bitten by snakes every year, and of these, 100,000 deaths occur annually, with 400,000 amputations and other severe health consequences (such as infection, tetanus, and scarring). Poor access to health care and scarcity of antivenoms increase the severity of the injuries and their outcomes. Snake venoms are a complex mixture of proteins, including metalloproteinase, serine protease, phospholipase A_2_, disintegrins, C-type lectins, myotoxins, and others [3, 4]. *Lachesis muta *(Bushmaster) is the longest venomous snake in the Americas and is distributed in the equatorial forests east of the Andes, ranging from eastern Ecuador, Colombia, Peru, northern Bolivia, and eastern and northern Venezuela, to Guyana, French Guyana, Surinam, and northern Brazil. Within their range, they are often abundant and are an important cause of snakebites [5]. *L. muta* snakebites are mainly characterized by systemic (generalized bleeding, coagulopathy, renal failure, and shock) and local effects (pain, hemorrhage, edema, and necrosis) [6, 7]. In South America, *B. jararaca *has a higher incidence of accidents (95%) than *L. muta* (2%), but, on the other hand, *L. muta* bites led to more severe symptoms (listed above) and have lethality indexes three times higher than *B. jararaca* [8]. Thus, reducing these indexes is necessary and must be fast.

Although antivenoms are effective against systemic effects, they do not inhibit the local effects. And, a few countries produce antivenom of adequate quality as well. Moreover, antivenoms may produce side effects and their cost of production may be expensive. In fact, worldwide the production of antivenoms is a global challenge. Due to all these limitations, the search for alternative or complementary treatment for snakebites deserves attention.

Plants are a rich source of pharmacologically active molecules that have been used for native people to treat several diseases, including snakebites. Several molecules have been isolated and tested against some lethal activities induced by snake venoms [9–11]. *Manilkara subsericea* (Mart.) Dubard (Sapotaceae), popularly known in Brazil as guracica, is an endemic species with edible fruits from the Brazilian Atlantic forest and grows in sandy coastal plains [12]. A chemical composition study of genus *Manilkara* has shown the presence of triterpenes [13, 14], saponins [15], and flavonoids [16]. The extracts of *Manilkara* displayed antimicrobial [13, 17, 18], antiparasitic [19, 20], anticholinesterase [21], against pest insects [22], or antitumoral [23] properties.

The present work investigates the ability of different ethanolic extracts of leaves and stem of *M. subsericea* and solvent-partitioned fractions to neutralize some *in vitro* and *in vivo* biological activities induced by *L. muta* snake venom.

## 2. Materials and Methods

### 2.1. Chemicals

All solvents were of the best analytical grade. Dimethylsulfoxide (DMSO) and azocasein were obtained from Sigma Chemical Co. (St. Louis, Missouri).

### 2.2. Plant Material and Extraction

*Manilkara subsericea *(Mart.) Dubard (Sapotaceae) was collected in January 2009 at the Jurubatiba Sandbank National Park, located in the Brazilian state of Rio de Janeiro (220° 16′ 4.93′′ S–41° 38′ 8.50′′ W), under license number 13659-2 from Instituto Brasileiro do Meio Ambiente e dos Recursos Naturais Renováveis—BAMA/SISBIO. The material was identified by Professor Dr. Marcelo Guerra Santos, a member of Department of Botany of State University of Rio de Janeiro (UERJ) and a voucher specimen was deposited in the Herbarium of UERJ and assigned under the number 13416.

The leaves (1.930 kg) and stems (0.960 kg) of *M. subsericea* were dried at 40°C for two days, ground, and macerated with ethanol 96% (v/v) at room temperature until exhaustion. Then, both materials were filtered and solvent evaporated using a rotary evaporator, yielding 530 g of ethanolic extract of leaves (ETL) and 169.3 g ethanolic extract of stems (ETS). These extracts were sequentially washed with solvents of increasing polarities (hexane and ethyl acetate). After complete removal of solvent under reduced pressure, using a rotary evaporator, 21.6 g hexane-soluble (HEL) and 11.5 g ethyl acetate-soluble (EAL) fractions from leaves and 12.1 g hexane (HES) and 13.7 g ethyl acetate (EAS) fractions from stems were obtained. All materials were dissolved in DMSO (30% v/v) to perform the biological assays.

### 2.3. Venom and Animals

*Lachesis muta* lyophilized venom was kindly supplied from Fundação Ezequiel Dias (FUNED), Brazil. Venom was diluted in physiological saline (1 mg/mL) and stored at −20°C until experiments. Male Swiss mice (18–20 g) were obtained from the Center of Laboratory Animals (NAL) of the Federal Fluminense University (UFF). All experiments were approved by the UFF Institutional Committee for Ethics in Animal Experimentation (protocol number 212) that were in accordance with the guidelines of the Brazilian Committee for Animal Experimentation (COBEA).

### 2.4. Proteolytic Activity

Proteolytic activity of *L. muta* venom was determined according to the method of Garcia et al. [24], using azocasein as substrate (0.2%, w/v, in 20 mM Tris-HCl, 8 mM CaCl_2_, and pH 8.8). Aliquots of *L. muta* venom (5–50 *μ*g/mL) were incubated with 0.4 mL azocasein at 37°C for 90 min in a total volume of 1.2 mL. The enzymatic reaction was stopped by adding trichloroacetic acid (5%, v/v, final concentration). The tubes were centrifuged at 15,000 ×g for 3 min. Then, the supernatant was removed and mixed with 2 N NaOH, and the tubes were read at A 420 nm (spectrophotometer Hitachi U-5100) to measure the release of azo dye and recorded from the samples. An effective concentration (EC) was defined as the amount of venom (*μ*g/mL) able to produce a variation of 0.2 units at A 420 nm. For the inhibitory experiments, one EC of *L. muta* venom (8.75 *μ*g/mL) was incubated at room temperature with the extracts or fractions of *M. subsericea* at 1 : 10 venom : plant (w/w). Then, proteolytic activity was determined accordingly. Positive control experiments were performed by incubating *L. muta* venom with saline or DMSO (1% v/v).

### 2.5. Clotting Activity

A pool of citrated normal human plasma (diluted with equal volume of saline) was obtained from the local blood bank (University Hospital Antônio Pedro of UFF) and was mixed with *L. muta* snake venom (2.5–70 *μ*g/mL) and the clotting time was monitored using an Amelung coagulometer, model KC4A (Labcon, Germany). The concentration of venom (*μ*g/mL) that clotted plasma in 60 seconds was considered as the minimum coagulant dose (MCD). To evaluate the inhibitory effect, the extracts or fractions of *M. subsericea* were incubated at room temperature for 30 minutes with one MCD of venom (25 *μ*g/mL) at 1 : 10 venom : plant (w/w), and then the mixture was added to plasma and clotting time was recorded. Negative control experiments were performed in parallel by mixing extracts or DMSO (0.5% v/v) with plasma in the absence of venom.

### 2.6. Hemolytic Indirect Activity

Hemolytic activity of *L. muta* venom was determined by the indirect hemolytic test using human erythrocytes and hen's egg yolk emulsion as substrate [25]. One minimum indirect hemolytic dose (MIHD) was defined as the lowest amount of *L. muta* venom (*μ*g/mL) able to produce 100% hemolysis. To verify the inhibitory action of *M. subsericea*, one MIDH (17.6 *µ*g/mL) was incubated at room temperature for 30 minutes with *M. subsericea* extracts or fractions at 1 : 10 venom : plant (w/w) ratio. Then, the hemolytic assay was performed. One hundred percent of hemolysis was obtained after lysing erythrocytes with distilled water. Negative control experiments were performed in parallel by mixing extracts or DMSO (1% v/v) with erythrocytes in the absence of venom.

### 2.7. Hemorrhagic Activity

Hemorrhagic lesions produced by *L. muta* crude venom were quantified using a procedure described by Kondo et al. [26], with minor modifications. Briefly, samples of venom (100 *µ*L) were injected intradermally (i.d.) into abdominal skin of mice. Two hours later, animals were euthanized and the skin was removed, stretched, and inspected for visual changes in the inner surface or subcutaneous layers to localize hemorrhagic spots. Hemorrhage was quantified as the minimum hemorrhagic dose (MHD), defined as the amount of venom (*μ*g/g) able to produce a hemorrhagic halo of 10 millimeters (mm) and that was the positive control. To evaluate the inhibitory effect, the extracts or fractions were incubated with two MHD (1.56 *μ*g/g) of venom at room temperature for 30 minutes at 1 : 10 venom : plant ratio (w/w). This mixture was injected i.d. into animals and hemorrhagic activity evaluated, as described above. In another set of experiments, *L. muta* venom was injected i.d. 15 min first or after injection of *M. subsericea* extract or fractions. Hemorrhage was expressed as the mean diameter (in millimeters) of the hemorrhagic halo induced by venom in the absence and presence of the extract. Negative control experiments were performed in parallel by injecting i.d. extracts or DMSO (1% v/v) in the absence of venom.

### 2.8. Edematogenic Activity

Edema-inducing activity of *L. muta* venom was determined according to Yamakawa et al. [27], with modifications. Groups of mice received subcutaneously (s.c.) 50 *μ*L of *L. muta* venom (0.74 *μ*g/g) in the right paw, while the left paw received 50 *μ*L of saline, DMSO (1% v/v), or extracts. One hour after injection, edema was evaluated as the percentage increase in weight of the right paw compared to the left one. Antiedematogenic activity was performed by incubating the extracts or fractions of *M. subsericea *with *L. muta *venom for 30 min at room temperature at 1 : 10 venom : plant ratio (w/w), and then mixture was injected s.c. into mice. Also, venom was also injected s.c. prior to or after the extract. Positive control experiments were performed by incubating *L. muta* venom with saline or DMSO (1% v/v) prior to injection.

### 2.9. Statistical Analysis

Results are expressed as means ± SEM of indicated number of animals or experiments performed. Student's *t*-test was used and *P* values of ≤ 0.05 were considered statistically significant.

## 3. Results

### 3.1. The *L. muta* Venom-Induced Proteolysis

**Figure 1:**
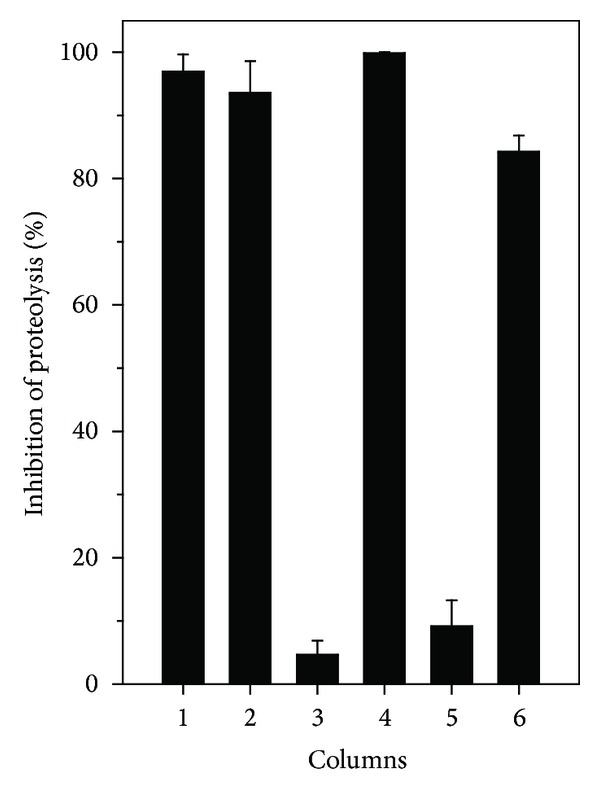
Effect of *M. subsericea* extracts on proteolysis induced by *L. muta* venom. *L. muta* venom (8.75 *μ*g/mL) was incubated with extracts of *M. subsericea* (87.5 *μ*g/mL) for 30 minutes; then proteolysis activity was measured as described in the Methods. Columns are *L. muta* mixed with (1) ethanolic extract of stem; (2) ethanolic extract of leaves; (3) hexane-soluble fraction of stem; (4) ethyl acetate-soluble fraction of stem; (5) hexane-soluble fraction of leaves; (6) ethyl acetate-soluble fraction of leaves. Data are expressed as means ± S.E.M of three individual experiments (*n* = 3).

The crude ethanolic extract of stem (ETS) and leaves (ETL) of *M. subsericea* (87.5 *μ*g/mL) inhibited 96% and 93%, respectively, the proteolytic activity of *L. muta *venom (8.75 *μ*g/mL) (Figure 1). Further, ETS and ETL were partitioned using two solvents of increasing polarities, hexane, and ethyl acetate, yielding hexane-soluble and ethyl acetate-soluble fractions, respectively. The ethyl acetate-soluble fraction of stem (EAS) inhibited 99% proteolysis induced by *L. muta *venom, whereas the hexane one (HES) inhibited 4%. And, ethyl acetate-soluble (EAL) and hexane-soluble (HEL) fractions of the leaves inhibited 84% and 9% proteolytic activity, respectively (Figure 1).

### 3.2. The *L. muta* Venom-Induced Clotting

**Figure 2:**
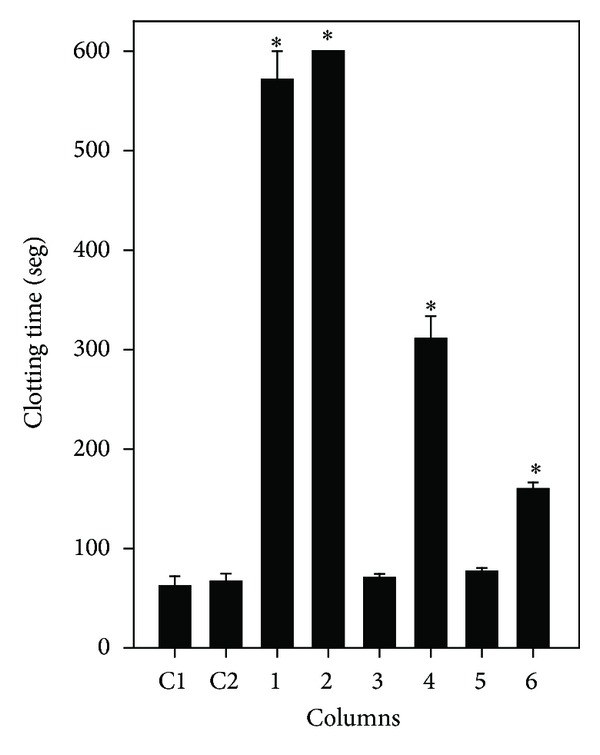
Effect of *M. subsericea* extracts on clotting induced by *L. muta* venom. *L. muta* venom (25 *μ*g/mL) was incubated for 30 minutes with *M. subsericea* extracts (250 *μ*g/mL); then mixtures were added to plasma and clotting monitored, as described in the Methods. Columns are *L. muta* venom incubated with NaCl (C1); with DMSO (C2), with ethanolic extract of stem (1) or leaves (2); with hexane- (3) or ethyl acetate- (4) soluble fractions of stem; with hexane- (5) or ethyl acetate- (6) soluble fraction of leaves. Data are expressed as means ± S.E.M of three individual experiments (*n* = 3). Significance level (*P* < 0.05) when compared to C1 or C2 columns. # means that plasma did not clot until 600 seconds of observation.

ETL (250 *μ*g/mL) fully prevented *L-muta*-induced clotting and ETS delayed clotting so that it took around eight times longer, when compared to control value (*L. muta* incubated with DMSO or saline) that clotted plasma in around 60 seconds (Figure 2). When venom was incubated with EAS or EAL, plasma clotted around 300 and 150 seconds, respectively (Figure 2). On the other hand, HES or HEL did not inhibit clotting induced by venom. DMSO (0.5% v/v, final concentration) did not interfere on clotting time induced by *L. muta* venom (Figure 2).

### 3.3. The *L. muta* Venom-Induced Hemolysis

**Figure 3:**
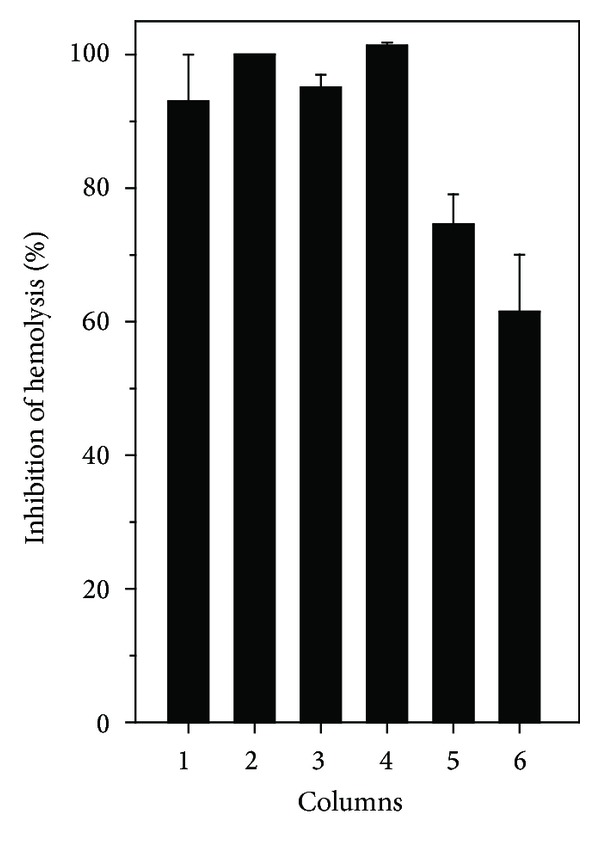
Effect of *M. subsericea* extracts on hemolysis induced by *L. muta* venom. *L. muta* venom (17.6 *μ*g/mL) was incubated with *M. subsericea* extracts (176 *μ*g/mL) for 30 minutes, and then hemolytic activity was performed. *L. muta* was incubated with ethanolic extracts of stem (1) or leaves (2); with hexane- (3) or ethyl acetate- (4) soluble fraction of stem; with hexane- (5) or ethyl acetate- (6) soluble fraction of leaves. Data are expressed as means ± S.E.M of three individual experiments (*n* = 3).

The extracts ETL or EAS (176 *μ*g/mL) inhibited 100% hemolysis induced by *L. muta* venom, while ETS or HES inhibited circa of 95% and HEL and ETL inhibited 75% and 60% hemolysis, respectively (Figure 3).

### 3.4. The *L. muta* Venom-Induced Hemorrhage

**Figure 4:**
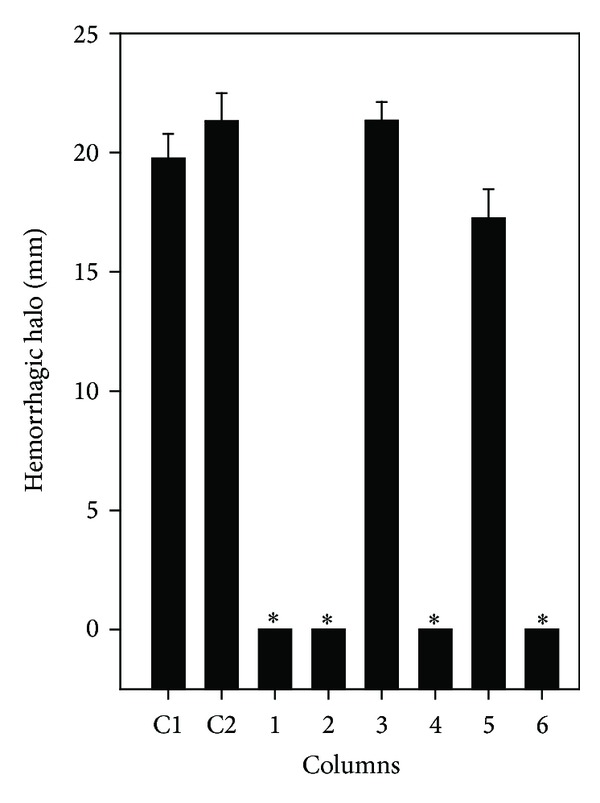
Effect of *M. subsericea *on hemorrhage induced by *L. muta* venom. *L. muta *venom (1.56 *µ*g/g) was incubated for 30 minutes with* M. subsericea *extracts (15.6 *μ*g/g); then mixture was injected into mice and hemorrhage evaluated, as described in the Methods. *L. muta* was incubated with saline (C1) or DMSO (C2); with ethanolic extracts of stem (1) or leaves (2); with hexane- (3) or ethyl acetate- (4) soluble fraction of stem; with hexane- (5) or ethyl acetate- (6) soluble fraction of leaves. Data are expressed as means ± S.E.M of three individual experiments (*n* = 4). Significance level (*P* < 0.05) when compared to C1 or C2 columns.

*L. muta* venom (1.56 *μ*g/g) injected into mice induced a hemorrhage halo of 20 mm that represents two MHD. But, in mice injected with venom mixed with ETL, ETS, EAS, or EAL (15.6 *μ*g/g), the mice were fully protected from hemorrhage induced by the venom (Figure 4). HEL inhibited the hemorrhage halo by 46% such activity and HES did not protect mice from *L. muta*-induced hemorrhage (Figure 4). However, when *L. muta* venom was injected into mice 15 minutes prior to or after ETS or ETL, protection was not observed anymore (results not shown).

### 3.5. The *L. muta* Venom-Induced Edema

**Figure 5:**
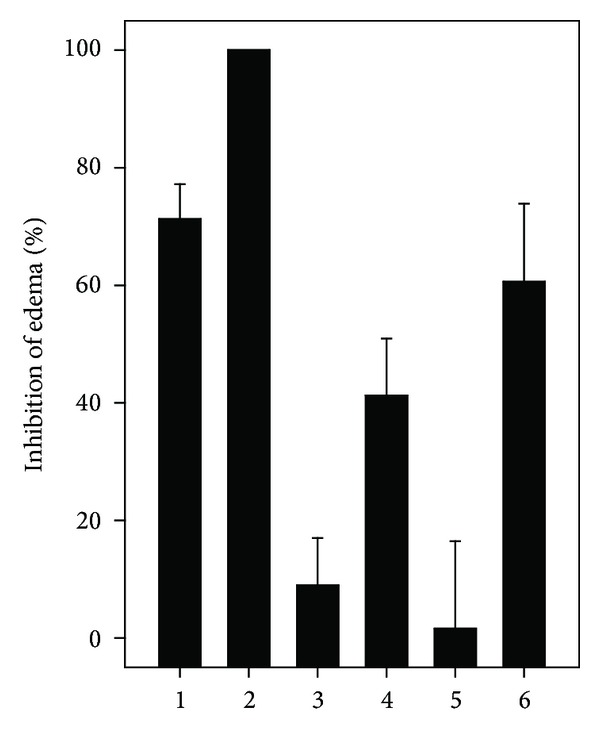
Effect of *M. subsericea *on *L. muta* venom-induced edema. *L. muta *venom (0.73 *μ*g/g) was incubated for 30 minutes with* M. subsericea *extracts (7.3 *μ*g/g); then mixtures were injected into mice and edema was evaluated, as described in the Methods. *L. muta* venom was incubated with ethanolic extracts of stem (1) or leaves (2); with hexane- (3) or ethyl acetate- (4) soluble fraction of stem; with hexane- (5) or ethyl acetate- (6) soluble fraction of leaves. Data are expressed as means ± S.E.M of three individual experiments (*n* = 4).

ETL (7.3 *μ*g/g) fully inhibited the formation of edema induced by *L. muta *venom (0.73 *μ*g/g), and ETS inhibited around 70% (Figure 5). HES and EAS inhibited edema below 5% and for EAS or EAL, 40 and 60% inhibition was observed. DMSO (1% v/v) neither interfered on hemorrhagic or edema of venom nor induced hemorrhage.

## 4. Discussion

Snakebites represent an important public health problem, mainly in rural and poor areas of developing countries, and because of that, they are considered neglected diseases according to World Health Organization. *L. muta* snakebites cause pain, edema, necrosis, hemorrhage, vascular disturbances, and death. Since the antiserum treatment is not so effective against local effects, it is necessary to search for alternative or complementary methods in order to efficiently neutralize such effects. Native people have used plants and their metabolites to treat several diseases, including snake bite [9], and the literature has proven their efficacy as well as their metabolites against some harmful activities of snake venoms [28].

Thus, in this work, we tested the ability of ethanolic crude extracts from stem and leaves of *M. subsericea* and also their soluble fractions (hexane-soluble and ethyl acetate-soluble) to inhibit some toxic and harmful activities induced by *L. muta* venom. Overall, extracts of the stem and leaves of *M. subsericea* as well as its fractions inhibited such activities. Their inhibition occurred around 90% the proteolytic activity of *L. muta *venom that has been associated with some symptoms that follow snakebites, as hemorrhage and/or clotting. As seen, extracts of stem or leaves inhibited clotting and hemorrhage induced by *L. muta* venom and thus would prevent bleeding or vascular disturbances caused by *L. muta* bites. ETL inhibited clotting more efficiently than ETS, but EAS was the most powerful to prevent clotting. In addition, ETS, ETL, EAS, and EAL inhibited 100% hemorrhage induced by *L. muta*. Hemorrhage induced by snake venoms may occur through activating blood clotting and/or degrading specific proteins (as fibrinogen) of the blood cascade system. Metalloproteases and serine proteases are responsible for such harmful effects. Data from the literature state that plants or their products, mainly those enriched in phenolic compounds [29], inhibit hemorrhage by chelating metal ions that are essential for the action of metalloproteases and serine proteases [9, 30, 31]. Other plants constituents, as flavonoids, xanthenes, and terpenoids, may also bind to the catalytic site of enzymes and, in turn, inhibit their enzymatic activity [32, 33]. Previous reports have identified components isolated from the root of *Hemidesmus indicus* [34] or from *Vitex negundo* and *Emblica officinalis* [35] able to neutralize lethal, hemorrhagic, clotting, and inflammatory activities of Viper venom, probably through an antioxidant action [36]. Phospholipases A_2_ promote several harmful activities, including hemolysis, platelet aggregation, edema, and myotoxicity [25, 37, 38], and as seen, *M. subsericea* interfered on some of these effects. ETS, ETL, HES, and HEL of *M. subsericea *inhibited 100% hemolysis, but only ETL fully protected mice from edema. ETS or EAL inhibited edema above 60% and EAS inhibited it by 40%. Polar and nonpolar secondary metabolites have been described as responsible for the inhibition of PLA_2_ in snake venom [39]. Besides plants, other natural products are inhibitors of PLA_2_ enzymes, such as diterpenes from the marine brown algae *Spatoglossum schröederi* [31] or *Canistrocarpus cervicornis* [40]. In addition, these algae also inhibited clotting and hemorrhagic activities induced by *L. muta* venom [31, 40]. Proteolysis, edema, and hemorrhage contribute to and/or are responsible for the local effects observed in victims after snakebite, which lead to amputation or morbidity in victims. Previous reports have indicated the presence of beta- and alpha-amyrin esters, such as their acetates, caprylates, and caproates in the hexanic extracts of leaves and stems [13, 22] that had antivenom properties. Moreover, spectroscopic analysis indicates the presence of some flavonoids, as quercetin, myricetin, and kaempferol-like in the ethyl acetate-soluble fraction of stems or leaves of *M. subsericea* (unpublished data). It should be mentioned that these substances displayed antivenom activity [9] and may be partially responsible for the inhibitory properties observed at the present study.

## 5. Conclusion

Since *M. subsericea* extracts inhibited *in vivo* (hemorrhagic and edematogenic) and *in vitro* (hemolytic, proteolytic, and clotting) harmful activities induced by *L. muta* venom, they may have a potential to the development of an alternative or complementary treatment for snakebites caused by *L. muta*.
